# Research on an Underwater Visual Enhancement Method Based on Adaptive Parameter Optimization in a Multi-Operator Framework

**DOI:** 10.3390/s26020668

**Published:** 2026-01-19

**Authors:** Zhiyong Yang, Shengze Yang, Yuxuan Fu, Hao Jiang

**Affiliations:** 1 Engineering Research and Design Institute of Agricultural Equipment, Hubei University of Technology, Wuhan 430068, China; hbut8740ysz@126.com (S.Y.); fyx15927030214@126.com (Y.F.); jh13713121596@126.com (H.J.); 2 Hubei Key Laboratory Modern Manufacturing Quality Engineering, School of Mechanical Engineering, Hubei University of Technology, Wuhan 430068, China

**Keywords:** underwater image enhancement, adaptive parameter optimization, multi-operator enhancement framework, feature robustness

## Abstract

Underwater images often suffer from luminance attenuation, structural degradation, and color distortion due to light absorption and scattering in water. The variations in illumination and color distribution across different water bodies further increase the uncertainty of these degradations, making traditional enhancement methods that rely on fixed parameters, such as underwater dark channel prior (UDCP) and histogram equalization (HE), unstable in such scenarios. To address these challenges, this paper proposes a multi-operator underwater image enhancement framework with adaptive parameter optimization. To achieve luminance compensation, structural detail enhancement, and color restoration, a collaborative enhancement pipeline was constructed using contrast-limited adaptive histogram equalization (CLAHE) with highlight protection, texture-gated and threshold-constrained unsharp masking (USM), and mild saturation compensation. Building upon this pipeline, an adaptive multi-operator parameter optimization strategy was developed, where a unified scoring function jointly considers feature gains, geometric consistency of feature matches, image quality metrics, and latency constraints to dynamically adjust the CLAHE clip limit, USM gain, and Gaussian scale under varying water conditions. Subjective visual comparisons and quantitative experiments were conducted on several public underwater datasets. Compared with conventional enhancement methods, the proposed approach achieved superior structural clarity and natural color appearance on the EUVP and UIEB datasets, and obtained higher quality metrics on the RUIE dataset (Average Gradient (AG) = 0.5922, Underwater Image Quality Measure (UIQM) = 2.095). On the UVE38K dataset, the proposed adaptive optimization method improved the oriented FAST and rotated BRIEF (ORB) feature counts by 12.5%, inlier matches by 9.3%, and UIQM by 3.9% over the fixed-parameter baseline, while the adjacent-frame matching visualization and stability metrics such as inlier ratio further verified the geometric consistency and temporal stability of the enhanced features.

## 1. Introduction

Underwater imaging technologies play a crucial role in acquiring detailed information about submarine environments and have been widely adopted in applications such as ocean exploration, biological research, and underwater robotics [1]. At present, the two primary approaches for underwater environment perception are acoustic sensing and optical vision. The former relies on sonar and other acoustic devices, offering advantages such as long detection range and strong penetrability, but it suffers from low resolution [2]. The latter typically employs underwater cameras or other optical sensors to obtain high-resolution images, making it well-suited for short-range object localization and environmental understanding [3].

**Figure 1:**
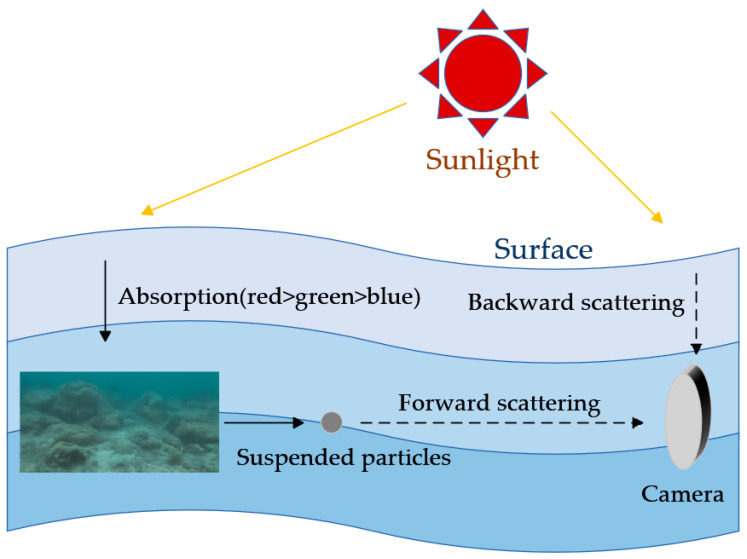
Underwater optical imaging model.

Vision-based underwater object detection and recognition are essential for enabling intelligent underwater robotic systems; however, the quality of underwater optical imaging is significantly degraded due to wavelength-dependent attenuation and path scattering [4], as illustrated in Figure 1. Different wavelengths of light exhibit varying attenuation rates in water: red light, which has the longest wavelength, attenuates the fastest, whereas blue and green wavelengths attenuate more slowly, resulting in the bluish–green color cast commonly observed in underwater images [5]. Meanwhile, suspended particles in the water induce both forward and backward scattering, which further reduces image contrast and blurs fine details [6]. These physical degradation phenomena often lead to color distortion, contrast reduction, and texture weakening in underwater images, severely impairing visual perception and subsequent high-level applications.

Extensive research has been conducted on underwater image enhancement and degradation compensation, particularly exploring image processing methods based on deep learning, physical models, and non-physical models [7]. Deep learning methods include end-to-end enhancement models based on CNN [8], GAN [9], and Transformer [10], as well as more recent learning-based approaches such as Ucolor [11] and DeepSeeColor [12], which can generate high-quality images in specific scenarios; however, their performance heavily depends on large-scale training data and computational resources, and they often exhibit limited generalization across different water domains and illumination conditions. Physical-model-based methods such as UDCP [13], GDCP [14], and Red Channel [15] restore image clarity by estimating the underwater attenuation model, but they rely on prior information such as water type, transmission estimation, and illumination direction. When these priors are unknown or vary significantly, color distortions are likely to occur. Non-physical-model-based methods such as HE [16], CLAHE [17], Retinex [18], and fusion-based method [19] do not require such priors and are computationally efficient. Nevertheless, their enhancement strength is controlled by fixed parameters, which often leads to over-enhancement, noise amplification, or color shifts in complex underwater environments. Overall, existing methods commonly suffer from reliance on a single type of degradation assumption, lack effective mechanisms for adaptive parameter tuning, and unstable performance across diverse scenes, making it challenging to maintain consistent visual quality and feature usability under different water conditions.

To address these issues, the main contributions of this study are as follows:

- A multi-operator collaborative enhancement pipeline tailored to the degradation characteristics of underwater optical images is proposed. This pipeline addresses various degradation effects caused by light absorption and scattering from three dimensions—luminance compensation, structural detail restoration, and color correction. It employs contrast-limited adaptive histogram equalization (CLAHE), a local contrast enhancement technique designed to improve image details under non-uniform illumination while mitigating over-enhancement and noise amplification, combined with highlight protection to suppress excessive local brightness enhancement; applies controlled unsharp masking (USM), a classical image sharpening technique for enhancing local contrast and edge details, together with texture gating to reinforce true structural details; and incorporates mild saturation compensation to alleviate bluish–green color bias, thereby producing enhanced images that appear more natural and are more suitable for feature extraction.
- An adaptive parameter optimization mechanism based on a unified scoring function is developed. By incorporating four constraints—ORB (Oriented FAST and Rotated BRIEF) feature gain, inlier matching gain, improvement in UIQM (Underwater Image Quality Measure), and latency penalty—a scoring scheme associated with real-time scene conditions is constructed. Using this scheme, global parameter search and real-time neighborhood refinement are performed for the three key operators—CLAHE clip limit, USM gain, and Gaussian scale—enabling the enhancement parameters to dynamically and robustly adapt to variations in water conditions, illumination, and degradation severity.
- Extensive experiments on public underwater datasets are conducted to evaluate both subjective visual quality and objective metrics. The superiority of the proposed enhancement method in terms of visual perception is validated on the EUVP and UIEB datasets. Quality indicators such as AG and UIQM are assessed on the RUIE dataset. The effectiveness of the adaptive multi-operator parameter tuning mechanism is verified on the UVE38K dataset, and its stability and consistency are further demonstrated through adjacent-frame matching visualizations and metrics including the two-hop survival ratio.

The remainder of this paper is organized as follows. Section 2 provides a detailed description of the proposed underwater image enhancement framework, including the multi-operator collaborative enhancement pipeline and the adaptive parameter adjustment mechanism. Section 3 presents the experimental design and evaluates visual quality, objective metrics, and dynamic adaptability across multiple public datasets. Section 4 concludes the paper and discusses potential directions for future research.

## 2. Methodology

### 2.1. Overall Framework

**Figure 2:**
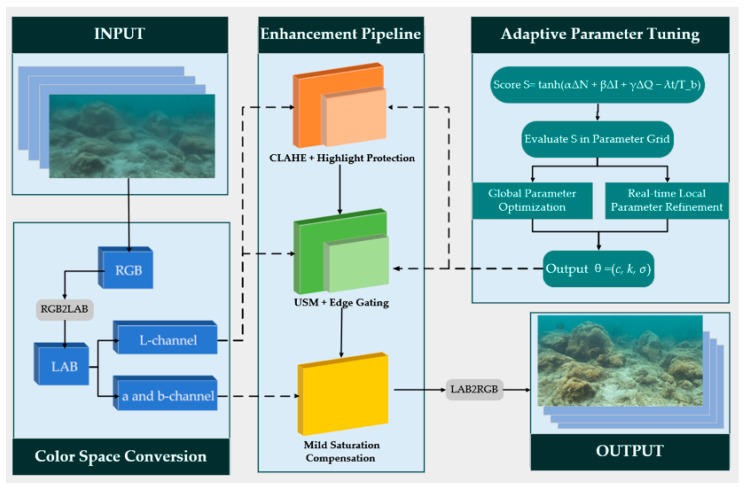
Overall framework diagram of underwater image enhancement based on adaptive parameter tuning mechanism.

The overall structure of the proposed underwater image enhancement framework is illustrated in Figure 2. First, the input underwater image is converted from the RGB color space to the LAB space, where the luminance channel (L) and chromatic channels (a and b) are separated, allowing brightness, structural, and chromatic information to be processed independently in different color domains. The enhancement pipeline then sequentially applies CLAHE together with highlight protection in the L channel to prevent excessive amplification in bright regions, followed by controlled USM with texture gating and threshold constraints to recover structural details while suppressing noise. Mild saturation compensation is applied in the ab channels to mitigate the bluish–green color bias caused by wavelength-dependent attenuation. Through this pipeline, image quality is comprehensively improved across the luminance, structure, and color dimensions.

To accommodate varying water conditions and scene dynamics, an adaptive parameter adjustment mechanism based on a unified scoring function is introduced. This mechanism performs offline global optimization and online neighborhood refinement of key parameters, including the CLAHE clip limit, USM gain, and Gaussian scale, enabling dynamic adjustment of the multi-operator enhancement parameters. Ultimately, the enhanced images produced by the framework exhibit more stable visual quality and feature usability, thereby providing a reliable input for subsequent vision-based tasks.

### 2.2. Image Enhancement Method

#### 2.2.1. Local Contrast Enhancement and Highlight Protection

In the luminance channel, this study introduces CLAHE into local regions to achieve contrast-limited histogram equalization [20], thereby enhancing contrast in low-luminance areas while preventing over-amplification in bright regions. Unlike global histogram equalization, it operates on small local regions rather than the entire image, enabling more effective enhancement of local details under non-uniform illumination conditions. By introducing a clip limit into the histogram redistribution process, CLAHE effectively suppresses noise amplification and over-enhancement in homogeneous regions.

Specifically, the luminance channel L is partitioned into 8 × 8 sub-blocks, and in each sub-block, a monotonic mapping function ${T_{k}}^{\left(c\right)}\left(\cdot \right)$ is constructed based on the clip limit *c*. Subsequently, bilinear interpolation is applied to the CLAHE output to obtain the locally enhanced result $L_{clahe}$ as defined in Equation (1):

(1) $$L_{clahe}\left(x,y;c\right)={CLAHE}_{c}\left(L\right)=bilinterp\left({T_{k}}^{\left(c\right)}\left(L\left(x,y\right)\right)\right),$$

A highlight protection factor is then constructed on $L_{clahe}$ to modulate the enhancement strength, preventing excessive stretching in bright regions. First, in underwater images, extreme luminance values are frequently caused by specular reflections, backscatter, or sensor noise. Using the maximum luminance value as a threshold would therefore be highly sensitive to such outliers and lead to unstable highlight suppression. Instead, using a high-percentile luminance value provides a robust estimate of the highlight threshold by attenuating the influence of isolated extreme values while preserving representative bright regions, therefore using the 97th percentile of the luminance distribution is computed to obtain the highlight threshold [21], as shown in Equation (2):

(2) $$\tau_{hi}={Perc}_{97}\left(L_{clahe}\right),$$

Next, a smooth protection function is defined based on the relative position of the pixel luminance with respect to the threshold [22], as expressed in Equation (3):

(3) $$m_{hi}\left(x,y\right)=smoothstep\left[L_{clahe}\left(x,y\right);\tau_{hi}-\varepsilon ,\tau_{hi}+\varepsilon \right],$$

where ε=1 controls the width of the smooth transition region [23]. The final highlight protection factor is defined in Equation (4):

(4) $$p_{rot}\left(x,y\right)=1-\lambda_{hi}m_{hi}\left(x,y\right),$$

where $\lambda_{hi}$ denotes the suppression coefficient for bright regions, and $p_{rot}$ represents the protection factor. By applying this factor to modulate the CLAHE output, the enhancement strength in high-luminance regions can be effectively suppressed, avoiding artifacts caused by excessive amplification while preserving the contrast enhancement effect of CLAHE in non-bright regions.

This luminance enhancement module provides a more stable luminance distribution for the subsequent structural enhancement and color correction steps and ensures more consistent enhancement performance across regions with different luminance levels.

#### 2.2.2. Controlled Sharpening and Edge Gating

In underwater imaging, scattering effects attenuate the high-frequency responses of true structural edges, while random noise can be erroneously amplified and misinterpreted as fine textures after CLAHE enhancement. Therefore, a controlled sharpening module [24] based on residual suppression and second-order gradient constraints is constructed on $L_{clahe}$, and a dual gating mechanism combining luminance and edge information is incorporated to achieve robust structural enhancement.

First, high-frequency residuals are extracted from $L_{clahe}$ to obtain structural details, and a luminance-based threshold is used to suppress noise-dominant regions. The residual map $d\left(x,y\right)$ and the residual mask $m_{d}\left(x,y\right)$ are defined in Equation (5):

(5) $$d\left(x,y\right)=L_{clahe}\left(x,y\right)-G_{\sigma }\;*\;L_{clahe}\left(x,y\right),\;m_{d}\left(x,y\right)=1\left\{\left|d\left(x,y\right)\geq \tau_{d}\right|\right\},$$

where $d\left(x,y\right)\;$ represents the high-frequency component of the CLAHE-enhanced luminance image, and $G_{\sigma }$ denotes a Gaussian kernel with standard deviation σ, which determines the spatial scale for separating meaningful structural components from high-frequency noise. The binary mask $m_{d}(x,y)$ is an indicator function: $m_{d}(x,y)=1$ when the residual magnitude ∣d(x,y)∣ exceeds the threshold $\tau_{d}$, and $m_{d}(x,y)=0$ otherwise. The threshold $\tau_{d}$ is used to suppress noise-dominant residual responses while preserving meaningful structural details. Next, the second-order gradient [25] is used to construct a noise-suppression term. The second-order gradient energy is defined in Equation (6):

(6) $$E\left(x,y\right)=\left|\nabla^{2}L_{clahe}\right|,$$

Its physical meaning lies in the fact that real edges produce stable second-order responses, while noise exhibits random fluctuations in the second-order gradient; thus, noise can be effectively suppressed through this term. To further limit excessive enhancement, a normalized and truncated energy map is constructed, as shown in Equation (7):

(7) $$\hat{E}\left(x,y\right)=clip\left(\frac{G_{\sigma }*E\left(x,y\right)}{{Perc}_{99}\left(G_{\sigma }*E\left(x,y\right)\right)};0,1\right),$$

An edge gating weight is then constructed using smoothstep [22], as defined in Equation (8):

(8) $$\omega_{tex}=smoothstep\left[\hat{E}\left(x,y\right);\theta_{lo},\theta_{lh}\right],$$

where $\theta_{lo}$ and $\theta_{lh}$ denote the lower and upper bounds of the smooth transition interval for the edge-gating function, defining the activation range of structural enhancement (these parameters were respectively set to 0.2 and 0.5 in the experiments). The smoothstep operator generates a continuous weight in the range [0, 1], enabling a gradual activation of enhancement in regions with sufficient structural energy while suppressing enhancement in texture-poor regions. This edge gating is combined with the highlight protection $p_{rot}\left(x,y\right)$ and the residual mask $m_{d}\left(x,y\right)$ to form the overall gain control. The combined gain function is given in Equation (9):

(9) $$g\left(x,y\right)=\omega_{tex}\left(x,y\right)\cdot p_{rot}\left(x,y\right)\cdot m_{d}\left(x,y\right),$$

Finally, the luminance sharpening output is defined in Equation (10):

(10) $$L_{out}\left(x,y\right)=L_{clahe}\left(x,y\right)+k\cdot g\left(x,y\right)\cdot d\left(x,y\right),$$

where k is the USM gain coefficient that regulates the overall strength of structural amplification, balancing detail enhancement and noise suppression. This parameter is optimized within a predefined range during the adaptive parameter optimization stage. $L_{out}$ is the sharpened luminance output. This controlled sharpening module achieves a balance between luminance protection, noise suppression, and edge response, effectively suppressing noise-induced artifacts in underwater imaging while enhancing true structural details.

#### 2.2.3. Mild Saturation Compensation

Due to the wavelength-dependent selective absorption of long wavelengths in underwater environments, images often exhibit an overall bluish–green color cast [26], even after the luminance channel has been restored using CLAHE and USM. To achieve natural color reproduction, a small-gain chromaticity adjustment is applied to the color channels (a,b) in the Lab space [27], as shown in Equation (11):

(11) $$\left\{\begin{array}{c} \hat{a}=\gamma_{S}\left(a-128\right);\hat{\;b}=\gamma_{S}\left(b-128\right)\\ a^{\prime }=clip\left(128+\hat{a},0,255\right);b^{\prime }=clip\left(128+\hat{b},0,255\right) \end{array}\right.,$$

where $\hat{a}$ and $\hat{b}$ are the gain-adjusted chromaticity values, $a^{\prime }\;$ and $b^{\prime }$ are the clipped chromaticity outputs, and $\gamma_{S}$ is the mild saturation gain coefficient [28]. The gain-adjusted chromaticity channels are then fused with the previously enhanced luminance channel $L_{out}$, followed by inverse color-space conversion to produce the final enhanced image, as defined in Equation (12):

(12) $$I^{\prime }=Lab2BGR\left(L_{out},a^{\prime },b^{\prime }\right),$$

### 2.3. Adaptive Parameter Tuning Mechanism

The underwater image enhancement method proposed above can effectively improve luminance layering, structural details, and chromatic balance. However, its core operator parameters—CLAHE clip limit c, USM sharpening strength k, and Gaussian smoothing scale σ—are fixed, which limits the ability to maintain consistent performance under varying water conditions, color casts, and changes in turbidity and illumination. Therefore, this study introduces an adaptive parameter optimization method that employs a unified scoring function integrating feature gains, geometric matching consistency, quality improvement, and latency constraints. This function guides both global parameter search and local refinement of the CLAHE clip limit, USM gain, and Gaussian scale, enabling dynamic adjustment of multiple operator parameters in response to scene variations.

**Figure 3:**
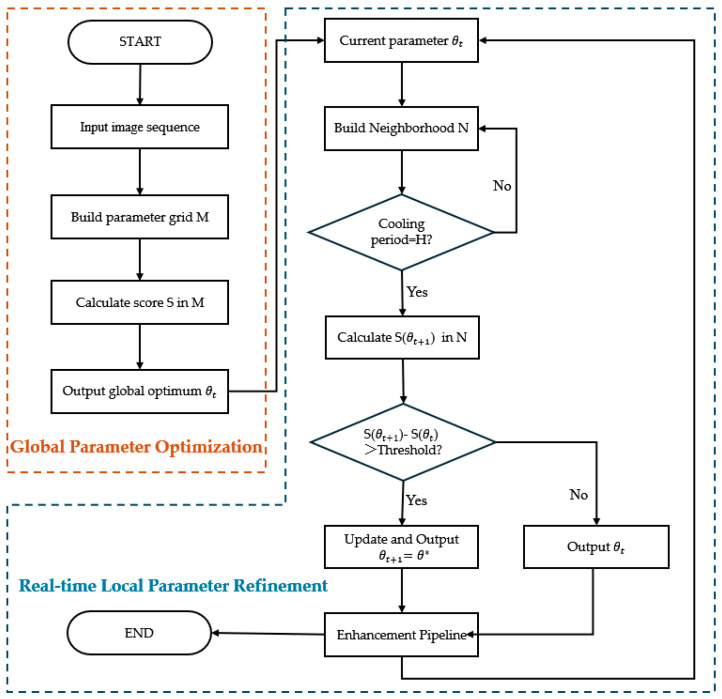
Process diagram of adaptive parameter tuning mechanism.

Figure 3 illustrates the process framework of the adaptive parameter tuning mechanism, which consists of two stages: global parameter optimization and real-time local parameter refinement. The former evaluates candidate parameter configurations within a predefined parameter grid to determine a robust initialization, while the latter updates parameters gradually during runtime based on neighborhood search strategies to accommodate changes in water conditions, illumination, and scene texture characteristics.

#### 2.3.1. Global Parameter Optimization

In this stage, a global search is performed over a predefined parameter grid to determine robust initialization parameters. The parameter vector is defined as θ=(c,κ,σ), where c denotes the CLAHE clip limit controlling brightness and contrast, k denotes the USM gain factor regulating sharpening strength, and σ denotes the Gaussian smoothing scale determining the structural response range. The candidate sets for each dimension are defined as C, K, and Z, respectively, forming a discrete parameter grid M. The Cartesian product of the three sets enumerates all candidate parameter combinations, as shown in Equation (13):

(13) M=C×K×Z,

where C={1.8,2.0,2.2,2.4}, K={0.4,0.6,0.8}, and Z={1.0,1.2}. These interval ranges were chosen based on the typical parameter settings reported in [17,25,29], covering enhancement strengths from mild to strong to ensure robustness and representativeness.

Let $I_{i}$ denote the i-th frame. A neighbor-pair set $P=\left\{\left(I_{i},I_{i+1}\right)\right\}$ was selected uniformly from a continuous sequence of images. For the raw image sequence (raw) and the enhanced sequence (enh), three relative improvements were defined: ORB feature gain ΔN, inlier match gain ΔI, and image quality gain ΔQ. Specifically, ORB features were extracted using the OpenCV implementation, and inlier correspondences were identified via RANSAC-based fundamental matrix estimation after descriptor matching. Image quality gain was computed on a frame-wise basis using the UIQM metric, and all relative gains were obtained by comparing enhanced images with the corresponding raw images under identical settings. These three terms are further mapped by tanh to obtain the gain term G(θ), as shown in Equations (14) and (15):

(14) $$∆N=\frac{N_{enh}-N_{raw}}{N_{raw}},∆I=\frac{I_{enh}-I_{raw}}{I_{raw}},∆Q=\frac{Q_{enh}-Q_{raw}}{Q_{raw}},$$

(15) $$G\left(\theta \right)=\alpha \tanh\left(\frac{∆N}{k_{1}}\right)+\beta \tanh\left(\frac{∆I}{k_{1}}\right)+\gamma \tanh\left(\frac{∆Q}{k_{2}}\right),$$

where α, β, and γ denote the preference weights for the three improvement terms and were fixed at 0.5, 0.3, and 0.2 in the experiments, respectively; Parameters $k_{1}$ and $k_{2}$ control the saturation ranges of the tanh functions and were set to 0.5 and 0.1, respectively [30]. Considering that the enhancement pipeline introduces additional processing time, the enhancement time $T_{enh}$ and its ratio to the time budget $T_{budget}$ are used to compute the delay ratio ΔT. By applying a delay penalty to G(θ), the final scoring function S(θ) is obtained, as defined in Equations (16) and (17):

(16) $$∆T=\frac{{{Perc}_{95}(T}_{enh})}{T_{budget}},$$

(17) $$S\left(\theta \right)=G\left(\theta \right)\cdot \exp\left(-\delta {∆T}^{r}\right),$$

where $T_{budget}$ denotes the predefined maximum allowable per-frame enhancement time, which was set to 45 ms in the experiments, δ is the delay penalty weight; and r = 1.2 represents the tolerance exponent, controlling the strength and curvature of the time-delay penalty. For each pair $(I_{i},I_{i+1})\in P$, the comprehensive score is computed for every parameter θ∈M. To evaluate the expected performance under an “average scene”, the scores over the pair set P are aggregated by averaging, as shown in Equation (18):

(18) $$\bar{S}\left(\theta \right)=\frac{\sum_{\left(I_{i},I_{i+1}\right)\in P}S_{i\to i+1}\left(\theta \right)}{\left|P\right|},$$

Finally, the parameter with the maximum average score is selected as the global optimal parameter $\theta_{t}$, as shown in Equation (19):

(19) $$\theta_{t}=arg\;\underset{\theta \in M}{\max}\bar{S}\left(\theta \right),$$

This global optimization stage provides a principled baseline for parameter initialization and serves as a warm-start for the subsequent online tuning mechanism, ensuring stability and convergence under complex and dynamic underwater scenes.

#### 2.3.2. Real-Time Neighborhood Refinement

Based on the globally optimal parameter $\theta_{t}$ obtained in the previous stage, this section introduces an adaptive local refinement mechanism during runtime to accommodate gradual variations in water turbidity, illumination, and texture distribution. For the current parameter $\theta_{t}$, a neighborhood set $N(\theta_{t})$ is defined. A local search is then performed within this neighborhood, and the scoring function S(θ) is evaluated to estimate the optimal parameter in real-time, as shown in Equations (20) and (21):

(20) $$Ν\left(\theta_{t}\right)=\left\{{(c}_{t}\pm ∆c,k_{t}\pm ∆k,\sigma \pm ∆\sigma )\right\},$$

(21) $$\theta *=\arg\underset{\theta \in Ν\left(\theta_{t}\right)}{\max}S\left(\theta_{t}\right),$$

where Δc, Δk, and Δσ are parameter step sizes defining the adjustment range for local refinement, and θ* denotes the optimal parameter within the current neighborhood.

To balance responsiveness and parameter stability, a cooldown interval H = 30 frames was set, such that neighborhood evaluation is performed once every H frames, and the parameter update is frozen until the next evaluation to avoid excessive oscillation. Additionally, a trigger condition is introduced before updating to ensure stable refinement: an update is performed only when $S^{\prime }\left(\theta *\right)-S^{\prime }\left(\theta_{t}\right)>\tau_{upd}$ and the cooldown interval has elapsed. The threshold $\tau_{upd}$ is estimated based on the distribution of score differences during the global search stage: specifically, the 75th percentile of the absolute score differences $\left|S^{\prime }\left(\theta *\right)-S^{\prime }\left(\theta_{t}\right)\right|$ is taken as the update boundary. The 75th percentile is adopted as a conservative update gate for the adaptive tuning process, such that only statistically significant score improvements trigger parameter updates, while minor fluctuations caused by noise or transient scene variations are suppressed. Compared with fixed absolute thresholds, this percentile-based criterion provides a data-driven and distribution-aware mechanism to balance adaptation responsiveness and temporal stability. Thus, the real-time parameter update rule is defined in Equation (22):

(22) $$\theta_{t+1}=\left\{\begin{array}{cc} \theta *, & S^{\prime \left(\theta *\right)}\geq S^{\prime \left(\theta_{t}\right)}+\tau_{upd}\\ \theta_{t}, & \;otherwise \end{array}\right.,$$

In summary, the real-time neighborhood refinement mechanism enables dynamic parameter adjustment in response to scene variations, complementing the global optimization stage. The global stage ensures robust initialization, while the refinement stage provides continuous adaptability during runtime.

## 3. Experimental Results and Analysis

### 3.1. Experimental Setup

The experiments in this study were implemented using Python 3.8.10 and open-source image processing libraries such as OpenCV 4.12.0, and were conducted on a system equipped with an Intel i7-12700 CPU and 16 GB of memory running Ubuntu 22.04. To verify the practicality and generalizability of the proposed enhancement method, all experiments were conducted under the same parameter configurations and hardware conditions.

### 3.2. Comparative Experiments on Enhancement Methods

To comprehensively evaluate the effectiveness of the proposed enhancement pipeline, experiments were conducted from two complementary perspectives: visual comparison and quantitative metric evaluation. The visual comparison focused on typical degraded underwater scenes in the EUVP and UIEB datasets, examining improvements in brightness, structural clarity, and color appearance produced by the enhancement method. The quantitative comparison, based on the RUIE dataset, evaluated standardized metrics to assess improvements in image sharpness, structural consistency, and overall visual quality. Together, these two experimental components complement each other and validate the effectiveness of the proposed method from both subjective and objective viewpoints.

#### 3.2.1. Visual Comparison on Public Datasets

The visual quality evaluation in this section uses two publicly available underwater image datasets: the EUVP dataset created by Islam et al. [31] and the UIEB dataset constructed by Li et al. [32]. Eight representative images covering diverse underwater scenes were selected for comparison. The proposed enhancement method was compared against several existing enhancement approaches, including Shades of gray [33], UDCP [13], WB_stretch, HE [16], CLAHE [17], and CLAHE + USM.

**Figure 4:**
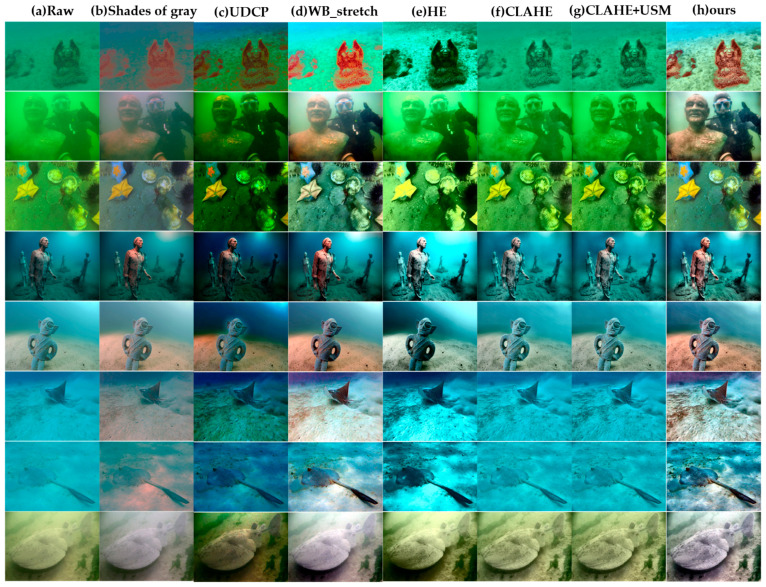
Qualitative comparison results of eight underwater images. From left to right: (**a**) Raw; (**b**) Shades of gray; (**c**) UDCP; (**d**) WB_stretch; (**e**) HE; (**f**) CLAHE; (**g**) CLAHE + USM; (**h**) ours.

As shown in Figure 4, Shades of gray [33] can remove part of the color cast, but its overall visual improvement is limited. UDCP [13] and WB_stretch can enhance brightness and contrast to a certain degree; however, they often introduce cold tones, resulting in bluish or greenish color shifts. HE [16] enhances brightness but tends to produce over-enhancement and color distortion. CLAHE [17] and CLAHE + USM both improved the brightness distribution and sharpness, but the latter may exhibit slight color shifts due to over-sharpening in bright regions. In contrast, the proposed method effectively suppresses noise and color bias while preserving structural details, resulting in an overall visual appearance that is more natural and closer to real underwater illumination conditions.

In summary, the visual comparison results indicate that the proposed method achieves more consistent and natural visual performance across various underwater scenes, providing higher-quality inputs for subsequent feature extraction and matching modules.

#### 3.2.2. Quantitative Evaluation on the RUIE Dataset

In this section, we evaluate the image quality using the RUIE dataset [34], which consists of three subsets: UCCS, UIQS, and UTTS. The UCCS subset provides various images with challenging color casts; the UIQS subset serves as a benchmark for assessing image enhancement performance under standardized conditions; and the UTTS subset contains underwater target images for evaluating detection-oriented enhancement methods [35]. Both qualitative and quantitative evaluations were conducted based on these datasets.

To objectively evaluate the effectiveness of the proposed method, quantitative experiments were conducted on the RUIE dataset. Specifically, 100 underwater images with diverse degradation characteristics were selected from the RUIE dataset, and commonly used objective image quality metrics, including Average Gradient (AG) [36], Perceptual Contrast Quality Index (PCQI) [37], Underwater Image Quality Measure (UIQM) [38], and Underwater Color Image Quality Evaluation (UCIQE) [39], were computed for each method. The results were then statistically averaged, and the corresponding quantitative comparisons are summarized in Table 1.

**Figure 5:**
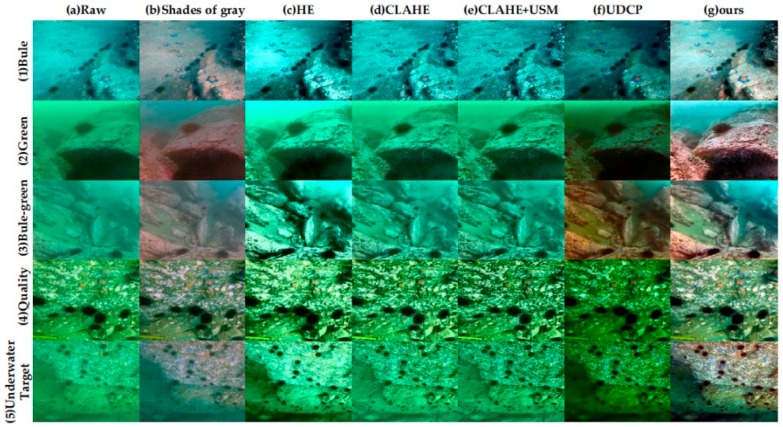
Visual comparison of five representative images selected from the RUIE dataset. From left to right: (**a**) Raw; (**b**) Shades of gray; (**c**) HE; (**d**) CLAHE; (**e**) CLAHE + USM; (**f**) UDCP; (**g**) ours. From top to bottom: (1) bluish-biased image; (2) bluish–green biased image; (3) greenish-biased image data in the UCCS dataset with different color biases; (4) underwater image quality data in the UIQS dataset that contains underwater images of various qualities for specific underwater mission and (5) underwater target mission data in the image dataset UTTS for a specific underwater mission.

As shown in Table 1, the proposed method achieved the highest scores on both AG and UIQM (0.5922 and 2.0895, respectively), indicating significant improvement in image sharpness, structural clarity, and overall perceptual quality while maintaining a stable and natural appearance. Although the proposed method showed a decrease in the UCIQE score (0.4367), it preserved the original color relationships more effectively in most scenes and avoided the color shifts caused by excessive enhancement. In contrast, HE achieved the highest UCIQE score (0.5024), but Figure 5 clearly shows that it introduced severe over-enhancement and color distortion. With respect to PCQI, the Shades of gray method achieved the highest score (0.9606), whereas the proposed method obtained a lower value (0.5185) compared with the other methods. This is mainly because the proposed enhancement process systematically reconstructs local contrast structures of the original image to emphasize structural details and improve feature discriminability. Since PCQI evaluates the consistency of contrast structures between enhanced and original images, significant changes to the original contrast relationships naturally lead to a decrease in PCQI values. Therefore, this result reflects differences in structural enhancement orientation rather than a degradation in image quality.

On this basis, to provide an intuitive visual illustration of the performance of different enhancement methods under typical degradation scenarios, five representative images were selected from the above image set for qualitative comparison, as shown in Figure 5. It presents a visual comparison between the proposed method and several representative underwater image enhancement approaches on the RUIE dataset [34]. The five experimental samples from top to bottom correspond to bluish images, greenish images, mixed bluish–green images, task-oriented underwater quality data, and underwater target-oriented data, respectively. From left to right, the comparison methods include Raw, Shades of gray [33], HE [16], CLAHE [17], CLAHE + USM, UDCP [13], and the proposed method (Ours). The proposed method effectively enhances underwater visibility, suppresses scene haze, preserves fine structural details, and maintains clearer edge information in target regions.

### 3.3. Validation of the Adaptive Parameter Tuning Mechanism

#### 3.3.1. Overall Performance Analysis

To comprehensively evaluate the effectiveness of the proposed adaptive parameter tuning mechanism, seven representative underwater sequences (turtle_2,mobula_4,marine_r10,marine_r8,marine_r3,marine_r2,coral_1) from the UVE38K dataset [40] were selected for experimentation. For each sequence, an initial segment was first processed using the global parameter optimization stage to determine a unified globally optimal initial parameter vector, which served as the starting configuration for the subsequent enhancement process. Based on this initialization, a comparative analysis was conducted between the proposed adaptive parameter tuning mechanism (Ours) and a fixed-parameter strategy (Baseline) to verify the practical role of the adaptive tuning mechanism under scene variations along video sequences. This comparison is explicitly formulated as an ablation study on the adaptive parameter optimization module.

Specifically, the proposed method (Ours) and Baseline shared an identical enhancement operator structure, image enhancement pipeline, and scoring function definition. Both methods were implemented within the same multi-operator collaborative enhancement framework, and their only difference was in the parameter update strategy. The Baseline method adopts the globally optimal initial parameter vector obtained via grid search in the global optimization stage and keeps the parameters fixed throughout the entire enhancement process for each sequence. In contrast, the proposed method dynamically updates the enhancement parameters based on the same optimal initialization through neighborhood search combined with scoring feedback, enabling adaptive parameter adjustment in response to scene variations along the video sequence.

Table 2 presents the results of executing the adaptive parameter tuning strategy (Ours) and the fixed-parameter strategy (Baseline) on each sequence. For each sequence, the table reports the optimal initial parameter vector, followed by the relative improvements of the number of ORB keypoints, the number of geometrically consistent inlier matches, and the image quality score t, denoted by ΔN, ΔI, and ΔQ, which are reported to quantify the performance gains of the adaptive strategy over the fixed-parameter baseline. As shown in Table 2, when both strategies started from the same optimal initial parameter vector, the adaptive tuning strategy achieved an average improvement of approximately 12.5% in ORB keypoint count, 9.3% in geometric inlier count, and 3.9% in the UIQM image-quality metric. These results demonstrate the effectiveness and robustness of the proposed adaptive optimization strategy in enhancing feature detectability, geometric consistency, and overall visual quality under diverse underwater conditions.

#### 3.3.2. Computational Efficiency Analysis

**Figure 6:**
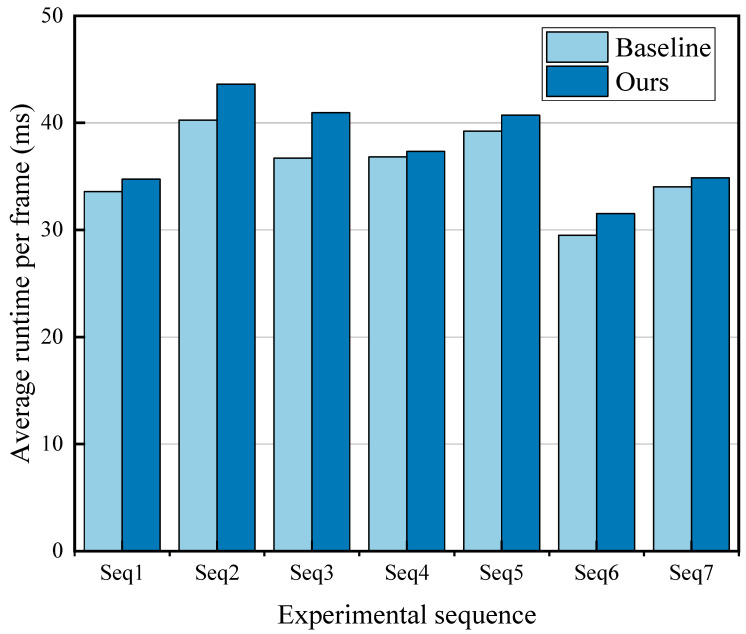
Average runtime per-frame comparison between the adaptive parameter tuning strategy (Ours) and the fixed-parameter baseline on seven sequences from the UVE38K dataset. From left to right: Seq 1-turtle_2; Seq 2-mobula_4; Seq 3-marine_r10; Seq 4-marine_r8; Seq 5-marine_r3; Seq 6-marine_r2; Seq 7-coral_1.

To evaluate the computational efficiency of the proposed adaptive enhancement framework, we further analyzed the runtime overhead introduced by the adaptive parameter tuning mechanism. Figure 6 presents a comparison of the average per-frame processing time between the fixed-parameter baseline strategy (Baseline) and the proposed adaptive parameter tuning mechanism (Ours) on seven representative sequences listed in Table 2.

As shown in Figure 6, the baseline strategy (Baseline) achieved an average processing time ranging from approximately 29 ms to 40 ms per frame, while the proposed method (Ours) operates within a range of approximately 31 ms to 44 ms per frame. In comparison, the adaptive tuning mechanism introduces an additional average overhead of approximately 2–4 ms per frame. Moreover, the adaptive parameter optimization is executed at a controlled interval and is further constrained by a score-based update threshold, as described in Section 2.3. These design choices effectively limit unnecessary parameter updates and prevent excessive runtime fluctuations. This runtime analysis demonstrates that the proposed adaptive enhancement framework achieves a good balance between performance improvement and computational cost, making it suitable for real-time or near-real-time underwater vision applications.

#### 3.3.3. Validation of Parameter Dynamics and Scoring Response

**Figure 7:**
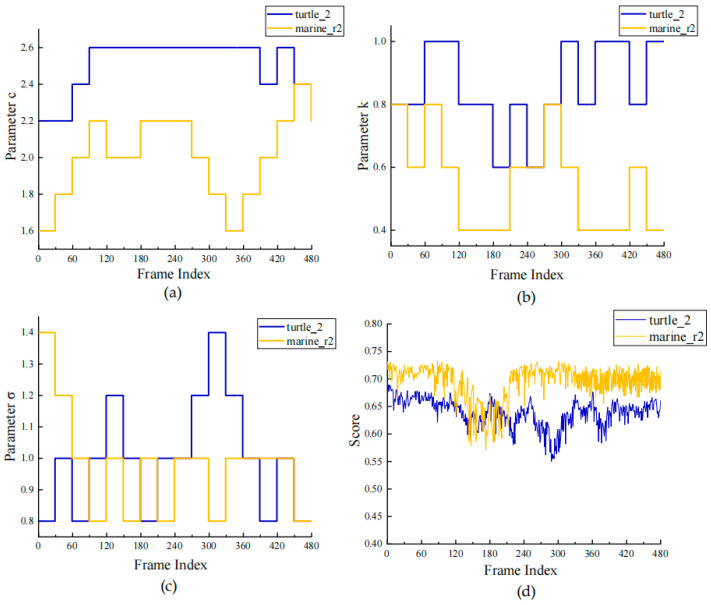
Trend chart of enhancing parameters c, k, σ and comprehensive scoring curve with frame sequence variation. (**a**) Trend of parameter c; (**b**) trend of parameter k; (**c**) trend of parameter σ; (**d**) trend of parameter comprehensive scoring curve.

To verify whether the proposed adaptive tuning strategy can achieve genuine dynamic responsiveness during runtime, this section conducts an analysis of parameter variation and scoring trends on two representative sequences, turtle_2 and marine_r2, selected from the UVE38K dataset [40]. Figure 7 illustrates the sequential variation in the enhancing parameters c, k, and σ, as well as the comprehensive scoring curves throughout the frame sequence.

From the parameter variations shown in Figure 7, it can be observed that all parameters adaptively adjust during sequential processing in response to changes in underwater illumination and environmental conditions. The turtle_2 sequence corresponds to a deep-water distant scene, where the overall illumination exhibits a pronounced bluish cast, and the distant region shows noticeable color attenuation and low contrast. Under such conditions, it is necessary to increase the contrast-enhancement parameter c and the sharpening weight k to compensate for brightness loss and color bias, while a relatively higher σ helps enhance edge clarity. However, strong enhancement operations may cause slight over-enhancement and amplified noise in local regions, leading to a lower final score. The marine_r2 sequence represents a mildly greenish underwater scene with relatively balanced brightness distribution and stable chromaticity. In this case, natural color restoration can be achieved within a lower parameter range, resulting in an average score of approximately 0.70. Overall, the amplitude of parameter variation reflects the strength of the adaptive response required for different environments, whereas the scoring curve reflects the quality and stability of the enhancement results.

#### 3.3.4. Feature Matching Visualization and Stability Metrics

To further verify the effectiveness of the proposed method in improving feature stability and matching quality from multiple perspectives, feature matching visualizations and stability metrics were analyzed across all test sequences.

Table 3 reports, for each sequence, the number of matched keypoints, the inlier ratio, the median Sampson error, and the two-hop survival ratio. From the table, it can be observed that the number of matched keypoints exceeded 3000 for most sequences, indicating that the enhanced images provide abundant and stable features across various scenes. The inlier ratio remained stable between 0.78–0.89, showing that the enhanced features exhibited strong geometric consistency during matching and did not easily result in large-scale mismatches. The Sampson error median serves as a geometric error indicator, where smaller values indicate more precise matches. Across the seven sequences, the median Sampson error lies between 0.16 and 0.33, indicating that the matched features obtained after enhancement were not only sufficient in quantity but also maintained relatively high matching accuracy. The two-hop survival ratio remained between 0.50–0.70, which demonstrates that the enhanced features can still maintain strong continuity under cross-frame propagation.

**Figure 8:**
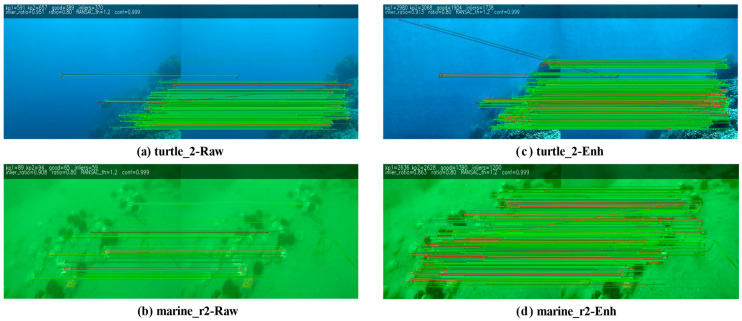
Matching visualization of turtle_2 and marine_r2 sequences. (**a**) turtle_2-Raw; (**b**) marine_r2-Raw; (**c**) turtle_2-Enh; (**d**) marine_r2-Enh.

Furthermore, this study selected the two sequences turtle_2 and marine_r2 above-mentioned and performed a visualization comparison of feature matching before and after enhancement. As shown in Figure 8. Matching visualization of turtle_2 and marine_r2 sequences. (a) turtle_2-Raw; (A) turtle_2-Enh; (b) marine_r2-Raw; (B) marine_r2-Enh, the number of feature correspondences increased significantly after enhancement, and the distribution became more uniform. In the bluish underwater scene turtle_2, enhancement yielded clearer textures on distant rocks, and the feature distribution expanded over a wider region. In the weak-texture greenish scene marine_r3, the algorithm balanced brightness suppression and structural enhancement, enabling the previously indistinct sandy bottom textures to be successfully identified. As a result, the matching correspondences became more continuous and stable across the entire image region. Combining the quantitative metrics and visualization results, it can be concluded that the proposed method consistently improved the feature density, geometric consistency, and temporal stability across various typical underwater scenes, providing more reliable feature inputs for subsequent vision-based SLAM front-end processing.

Despite the stable performance observed in most typical underwater scenarios, the proposed method still exhibits limitations under certain challenging conditions. Since the enhancement framework is primarily constructed upon traditional image processing operators, its behavior essentially relies on redistributing brightness, contrast, and structural details. In underwater environments involving artificial light sources or reflective surfaces, strong local highlights, glare, and severely non-uniform illumination may occur. Such degradations are characterized by extreme local intensity stretching rather than simple global brightness shifts. Under these conditions, enhancement strategies based on thresholding or percentile statistics are required to balance highlight suppression and dark-region detail recovery. When luminance distributions become excessively extreme, a single-parameter adjustment may struggle to simultaneously satisfy both objectives, potentially leading to insufficient local structure restoration or unstable structural responses. Although the proposed method introduces highlight suppression and constrains enhancement strength to mitigate these effects in common scenarios, performance degradation may still arise when high-intensity regions dominate the scene or illumination conditions change abruptly. These failure cases highlight the need for more adaptive and context-aware enhancement strategies, which will be explored in future work.

## 4. Conclusions and Future Work

### 4.1. Conclusions

(1) This paper proposes an underwater image enhancement method tailored for diverse aquatic environments. By integrating brightness enhancement, structural detail recovery, and chromaticity compensation into a collaborative multi-operator pipeline, the method effectively addresses degradation phenomena caused by underwater light absorption and scattering, including brightness attenuation, structural weakening, and color shifts.
(2) To further improve the robustness of the enhancement pipeline under varying water conditions, this paper introduces an adaptive parameter optimization mechanism based on multi-metric evaluation. Through offline global parameter search and online neighborhood refinement, key parameters including the CLAHE clip limit, USM gain, and Gaussian scale are dynamically adjusted according to scene variations, thereby overcoming the limitations of fixed-parameter strategies in multi-scene environments.
(3) Experimental results on public datasets such as RUIE demonstrate that the proposed method achieved the best performance in structural sharpness (AG = 0.5922) and overall visual quality (UIQM = 2.0895). On the UVE38K multi-sequence benchmark, compared with fixed-parameter baselines, the proposed approach yielded an average improvement of 12.5% in ORB keypoint count, 9.3% in inlier matches, and 3.9% in UIQM, while also exhibiting higher geometric consistency and temporal stability in correspondence visualization and inlier-ratio analysis. These results validate the effectiveness of the proposed method in improving both visual quality and feature stability, thereby providing more reliable input images for vision-based SLAM front-end processing.

### 4.2. Future Work

Although the proposed method demonstrated stable performance across diverse underwater conditions, several limitations remain and motivate future research directions. First, the current enhancement pipeline primarily relies on traditional image-processing operators and manually designed parameter optimization rules. While this design ensures efficiency and interpretability, it may limit adaptability under highly complex or previously unseen underwater environments. A promising direction for future work is to incorporate learning-based or hybrid approaches for adaptive parameter estimation. For example, lightweight deep models may be introduced to learn underwater optical properties and scene distributions, enabling more accurate structural restoration and color correction. In addition, learning-based or reinforcement-learning strategies could be explored to replace or complement the manually designed scoring function, allowing for more flexible and data-driven parameter adjustment while maintaining interpretability. Future studies will investigate its performance in real-world deployments, where enhanced visual inputs may support more stable three-dimensional reconstruction and improve the robustness of SLAM front-end feature tracking. Finally, further research may investigate temporal consistency within enhanced sequences, constructing cross-frame constraints to strengthen geometric continuity and structural stability.

## Figures and Tables

**Table 1:** Average quantitative evaluation results on 100 underwater images from the RUIE dataset (The best performance for each metric is highlighted in bold).

Method	AG [34]	PCQI [35]	UIQM [36]	UCIQE [37]
Raw	0.2102	0.9974	1.0449	0.3649
Shades of gray [31]	0.2337	**0.9606**	1.1643	0.2821
HE [14]	0.4038	0.6428	1.9616	**0.5024**
CLAHE [15]	0.4100	0.7285	1.3717	0.4182
CLAHE + USM	0.5281	0.5997	1.6224	0.4607
UDCP [11]	0.2651	0.7231	1.9641	0.4506
Ours	**0.5922**	0.5185	**2.0895**	0.4367

**Table 2:** Quantitative comparison between the adaptive parameter tuning strategy (Ours) and the fixed-parameter baseline (Baseline) on seven sequences from the UVE38K dataset. (The ↑ indicates relative improvement, where higher values represent better performance).

Sequence	Category	Best Initθ	Kp_enh	I_enh	Q_enh	Relative Improvement		
			ΔN	ΔI	ΔQ	ΔN ↑	ΔI ↑	ΔQ ↑
turtle_2	Ours	(2.4, 0.8, 1.0)	1.193	1.023	0.515	7.3%	4.2%	0.7%
	Baseline		1.112	0.981	0.511			
mobula_4	Ours	(1.8, 0.4, 1.2)	0.554	0.464	0.042	22.2%	14.9%	2.4%
	Baseline		0.454	0.404	0.041			
marine_r10	Ours	(2.2, 0.8, 1.0)	4.379	3.035	0.538	13.6%	8.4%	7.2%
	Baseline		3.856	2.798	0.502			
marine_r8	Ours	(2.0, 0.8, 1.2)	12.730	9.017	0.822	20.6%	20.6%	5.7%
	Baseline		10.555	7.477	0.778			
marine_r3	Ours	(1.8, 0.8, 1.2)	6.368	4.860	0.815	8.0%	6.7%	10.1%
	Baseline		5.895	4.552	0.740			
marine_r2	Ours	(1.8, 0.8, 1.2)	21.295	15.495	1.025	10.3%	6.4%	1.7%
	Baseline		19.298	14.569	1.008			
coral_1	Ours	(2.2, 0.8, 1.0)	0.332	0.252	0.002	5.7%	4.1%	0%
	Baseline		0.314	0.242	0.002			
Overall Average Improvement						12.5%	9.3%	3.9%

**Table 3:** Stability evaluation of seven sequences from the UVE38K dataset.

Sequence	Feature Matching and Stability Metrics			
	Matched Keypoints	Inlier Ratio	Sampson Error Median	Two-Hop Survival Ratio
turtle_2	3991	0.8251	0.2535	0.5432
mobula_4	3399	0.8914	0.1662	0.7055
marine_r10	3477	0.8160	0.3064	0.5366
marine_r8	2458	0.7843	0.2981	0.4998
marine_r3	3385	0.8143	0.2918	0.5305
marine_r2	2053	0.7820	0.2830	0.5079
coral_1	14,052	0.8515	0.2555	0.5696

## Data Availability

The data presented in this study are available from the corresponding author upon reasonable request. The data are not publicly available due to privacy restrictions.
